# Exploring Cancer Prevention Challenges for People with Intellectual Disabilities: Perspectives from Family Caregiver

**DOI:** 10.3390/healthcare12232463

**Published:** 2024-12-06

**Authors:** Špela Golubović, Dragana Simin, Vladimir Vuković, Semra Demirović, Dragana Milutinović

**Affiliations:** 1 Department of Special Education and Rehabilitation, Faculty of Medicine, University of Novi Sad, 21000 Novi Sad, Serbia; 2 Department of Nursing, Faculty of Medicine, University of Novi Sad, 21000 Novi Sad, Serbia; dragana.milutinovic@mf.uns.ac.rs; 3 Institute of Public Health of Vojvodina, 21000 Novi Sad, Serbia; vladimir.vukovic@mf.uns.ac.rs; 4 Department of Epidemiology, Faculty of Medicine, University of Novi Sad, 21000 Novi Sad, Serbia; 5 Udruženje za Pomoć Mentalno Nedovoljno Razvijenim Osobama, 36300 Novi Pazar, Serbia

**Keywords:** intellectual disabilities, cancer prevention, caregivers, healthcare barriers, mixed methods, Bronfenbrenner’s theory

## Abstract

**Background:** Cancer prevention is a crucial public health strategy, with 30–50% of cancers being preventable through early screening and lifestyle modifications. However, people with intellectual disabilities (PWID) face significant barriers to cancer prevention services, leading to delayed diagnoses and poorer outcomes. Family caregivers play a key role in bridging the healthcare access gaps for PWID, but there is limited research exploring their perspectives on cancer prevention. This study examined the needs, challenges, and strategies family caregivers employ in cancer prevention for PWID, framed within Bronfenbrenner’s ecological systems theory. **Methods:** A mixed methods sequential explanatory design was used. Quantitative data were collected through a structured questionnaire from 41 caregivers of PWID, followed by qualitative semi-structured interviews with 15 caregivers to explore the quantitative findings further. Data were analysed using descriptive statistics, chi-square tests, and thematic content analysis. **Results:** The study revealed that 75.6% of caregivers had not received information about cancer prevention for PWID, and 63.4% did not regularly coordinate cancer screenings. Barriers identified included a lack of accessible information, logistical challenges, and emotional strain. However, 80.5% of caregivers expressed a need for additional training to support their roles in cancer prevention. Qualitative findings highlighted four key themes: promoting a healthy lifestyle, access to healthcare services, health literacy, and psychosocial support. **Conclusions:** The findings emphasise the need for tailored cancer prevention strategies and educational resources for caregivers of PWID. Addressing these gaps requires systemic changes in healthcare practices, better coordination of services, and enhanced support for caregivers to reduce the barriers to cancer prevention for PWID.

## 1. Introduction

Over the past decade, the global burden of cancer has ranged from 0.6% to 3% of the population, depending on the cancer type, with a notable reduction in mortality rates averaging around 1.6% [1,2,3]. Despite these improvements, cancer remains a leading cause of death worldwide; however, it is estimated that 30–50% of cancers can be prevented through early screening and preventive strategies, which are critical in reducing cancer incidence and improving survival rates [1]. These prevention efforts are particularly vital for vulnerable populations, such as people with intellectual disability (PWID), who face significant barriers to healthcare access and cancer prevention services. According to the American Association on Intellectual and Developmental Disabilities, intellectual disabilities are characterised by intellectual functioning limitations, including reasoning, learning, and problem solving. Additionally, individuals may experience challenges in everyday social and practical skills, such as language and communication, interpersonal skills, social responsibility, and activities of daily living. These limitations typically arise any time before a person turns 22, including during the prenatal period [4].

Research consistently shows that people with ID are underserved in routine cancer screening programs, leading to delayed diagnoses and poorer health outcomes [5,6,7]. For example, cancer is reported as a cause of death approximately 1.5 times more often in PWID compared to the general population [8]. The low uptake of cancer screening within this population can be attributed to multiple factors, including communication challenges, fear, anxiety, and a lack of accessible healthcare [6,9]. These barriers often result in missed opportunities for early detection and timely intervention, further exacerbating health disparities for PWID [10,11]. Additionally, healthcare professionals may lack the necessary training to provide tailored care. They may not recognise or effectively manage complex health conditions prevalent in PWID, leading to disparities in preventive care [12,13].

Addressing these gaps requires a concerted effort from healthcare professionals, caregivers, and policymakers to ensure that cancer prevention initiatives are inclusive and accessible. Due to reduced funding for government services, PWIDs often rely on informal, unpaid assistance from their social networks [14]. Families play a crucial role in providing and coordinating various forms of informal support, including emotional, practical, and social assistance. 

Family caregivers are critical in bridging these healthcare gaps [15]. As part of the “microsystem” in Bronfenbrenner’s ecological systems, family members are directly involved in the day-to-day health management of PWID [16]. Recent studies confirm the importance of family involvement, showing that caregivers are pivotal in ensuring adherence to health recommendations and promoting cancer prevention strategies tailored to the needs of individuals with cancer [17].

Family caregivers often advocate for timely medical care, ensuring that individuals with ID receive the necessary cancer screenings and follow-up care. They also ensure access to cancer screenings and provide emotional support, helping PWID navigate challenging healthcare systems. Additionally, caregivers offer essential emotional support that can help reduce the fear and anxiety PWID may experience during medical procedures, thus increasing their willingness to participate in cancer prevention activities [7]. Without this family involvement, PWID would face even greater barriers to engaging in preventive healthcare.

In the broader context of Bronfenbrenner’s ecological framework, family caregivers’ roles intersect with other systems, such as the healthcare system (mesosystem) and healthcare policies and infrastructure (exosystem). Namely, studies have shown that family members coordinate care between different healthcare professionals and navigate systemic barriers such as transportation challenges and fragmented care [18,19]. Furthermore, at the “macrosystem” level, societal attitudes and policy frameworks influence the availability and accessibility of cancer prevention services for PWID, highlighting the need for inclusive public health strategies that support caregivers in their crucial role. According to Burke et al. [20], inclusive public health strategies must recognise and support family caregivers of PWID, address their health needs, and ensure access to necessary services and resources.

Recent research underscores the importance of cancer prevention strategies tailored to the unique needs of PWID [21]. Health-aware family caregivers are vital in improving cancer prevention outcomes and overall health for PWID by addressing the systemic and interpersonal barriers to healthcare access. However, the practical application of these insights requires targeted interventions, including caregiver training programs, policy reforms, and improvements in healthcare practices. These interventions aim to overcome communication barriers by utilising appropriate augmentative technologies. Additionally, providing clear guidelines for presenting screening and examination procedures is essential to address cognitive challenges [20,21]. It is also important to respect universal design principles to ensure that spaces and devices are specifically suited to meet the needs of this population.

Therefore, this mixed methods study aimed to explore the needs of family caregivers in cancer prevention for PWID. It focuses on understanding caregivers’ unique challenges and identifying strategies to improve cancer prevention efforts framed within Bronfenbrenner’s ecological systems theory. By addressing these challenges, the study seeks to contribute to developing more inclusive and supportive public health strategies that prioritise the needs of individuals with intellectual disabilities and their caregivers.

## 2. Materials and Methods

### 2.1. Study Design

This study utilised a mixed methods research design, employing a sequential explanatory approach across two phases. The sequential explanatory design was selected first to gather and analyse quantitative data, followed by qualitative data collection to provide a more in-depth exploration and explanation of the quantitative findings [22]. This approach was chosen over concurrent methods because it allows for a structured exploration of patterns that emerge from the quantitative data and enables the qualitative phase to target areas where more explanation is needed specifically. The integration of the quantitative and qualitative phases occurred during the interpretation of results, where themes from the qualitative interviews were used to explain and deepen the understanding of patterns found in the quantitative analysis.

### 2.2. Sample, Setting and Data Collection

The sample included 41 caregivers of PWID (parents n = 35/85.4%, siblings n = 5/12.2% and guardian n = 1/2.4%). The individuals they care for are adults with intellectual disabilities (ID), of which 20 (48.85%) are men and 21 (51.2%) are women. In terms of age, the structure of these individuals is as follows: under 18 years old, there are 15 (36.6%); between 18 and 30 years old, there are 11 (26.8%); between 31 and 45 years old, there are 10 (24.4%); and between 45 and 60 years old, there are 5 (12.2%). Regarding the level of intellectual disability, the structure is as follows: with mild ID, there are 7 (17.1%); with moderate ID, there are 18 (43.9%); with severe ID, there are 6 (14.6%); and with profound ID, there are 10 (24.4%).

All caregivers were personally contacted and informed about the study with the assistance of staff members from adult day-care centres for PWID in Serbia. These centres, often non-governmental organisations, provide occupational and work activities for PWID during the day after the participants return to their families.

PWID family members were first informed about the research and asked if they wished to receive more details. If they agreed, one of the researchers interviewed them face to face, explaining the purpose and methods of the study and the process of completing the questionnaire and participating in the follow-up interview. The questionnaire and interview were conducted in adult day-care centres that provide service and support to PWID. They were arranged with the parents at a time that was most convenient for them. One of the manuscript’s authors, SD, was always present during the completion of the questionnaire. The details regarding the conduct of the interview are described in the following text.

#### 2.2.1. Quantitative Phase

The questionnaire “Cancer Prevention Strategies for People with Intellectual Disabilities” was designed for this study to assess caregivers’ knowledge, attitudes, and experiences regarding cancer prevention. The questionnaire was divided into six sections (general information about the person with ID, attitudes and beliefs, preventive activities, education and support, barriers and challenges, and support and resources), comprising 26 items. Responses were collected by selecting answers or rating their level of agreement with statements using a five-point Likert scale, ranging from 1 (strongly disagree) to 5 (strongly agree). The questionnaire was piloted with a small sample of caregivers before the full study, and minor adjustments were made based on their feedback.

#### 2.2.2. Qualitative Phase

In the second phase, a qualitative analysis approach was used, employing semi-structured interviews—the qualitative phase aimed to understand better caregivers’ needs and priorities related to cancer prevention strategies. The interview guide was developed based on the questionnaire findings, focusing on exploring areas that required further elaboration, such as which strategies caregivers had tried, what had worked or not worked, and why they believed certain outcomes occurred. The qualitative elements of the study adhered to the Consolidated Criteria for Reporting Qualitative Studies (COREQ) [23].

The principal investigator (SG), a professional with a special education and rehabilitation background, conducted the interviews. Each interview was completed within a 30-min and 50-min time frame and was conducted face-to-face. The coding process involved multiple rounds of reading and categorising interview transcripts, reviewed independently by two researchers (DM and DS), both experienced in qualitative research and nursing. Inter-rater reliability was assessed, and any discrepancies in coding were resolved through discussion until a consensus was reached. A sample of 15 mothers agreed to participate and completed the interview. This sample size is considered sufficient based on recommendations for qualitative research [24].

The quantitative and qualitative data integration occurred in the final analysis stage. The qualitative results were used to explain and expand on the findings from the quantitative phase, specifically addressing key themes that emerged from the questionnaire, such as barriers to cancer prevention and caregiver support needs.

### 2.3. Statistical Analysis

Quantitative data analysis was performed using descriptive and inferential statistics. Attributive characteristics are presented by absolute and relative frequency, while the chi-square test of independence (χ²) was used to determine the significance of the differences. In addition, effect size (Cramer’s V) was calculated to quantify the obtained differences. Statistical processing and analysis of the obtained results were performed using the software package IBM SPSS 28 Statistics, and all tests were two-sided with a significance level of *p* < 0.05. Qualitative processing included thematic content analysis [25].

### 2.4. Ethical Considerations

All procedures performed in human participant studies followed the institutional and/or national research committee’s ethical standards, the 1964 Helsinki Declaration, and its later amendments or comparable ethical standards. Approval was obtained from the Faculty of Medicine Commission for the Ethics of Clinical Research, the University of Novi Sad, Serbia 01-39/227/1 of 15 April 2024.

All caregivers gave informed consent before participating in the study and were informed of their right to withdraw from the study at any time. Additionally, all participants were offered support and counselling services following the completion of the study.

## 3. Results

In order to examine caregivers’ knowledge, attitudes, and experiences regarding cancer prevention, the data obtained from the questionnaire were analysed.

The results from Table 1 highlight several critical areas where families of PWID face challenges regarding cancer prevention. Namely, 75.6% of caregivers reported not receiving information about cancer prevention for PWID. This finding suggests a critical gap in healthcare communication and educational outreach, pointing to a need for more accessible, targeted information to be disseminated to caregivers. On the contrary, only 24.4% of caregivers indicated that they had received such information, indicating that many caregivers may lack the necessary knowledge to support cancer prevention efforts effectively.

Regarding the need for additional training, 48.8% of caregivers agree, and 31.7% of caregivers completely agree that such training would help improve cancer prevention in PWID. These results indicate that caregivers are open to and see the value of further education to better support their family members with ID. In addition, 19.5% was neutral, suggesting that while some caregivers may not yet recognise the need for training, the majority see it as beneficial.

Alarmingly, 63.4% of caregivers reported that they do not regularly plan or participate in organised cancer screening programs (e.g., mammography, colonoscopy, gynaecological exam) for their family members with ID. These data underscore healthcare systems’ need for greater support and encouragement to ensure regular, proactive screenings.

When it comes to the difficulties encountered during regular cancer screenings for their family members with ID, it was determined that only 24.4% of caregivers never experienced them. In contrast, the others encountered such difficulties with varying frequency (12.2% to 26.8%). The abovementioned highlights the need for more comprehensive support and accommodations within healthcare systems to ensure that PWID can easily access regular screenings.

Satisfaction with the support from healthcare professionals was diverse. While some caregivers were satisfied with the support they received (31.8%), almost half of caregivers (41.5%) were neutral, which might reflect a lack of meaningful interactions or ambivalence about the support received. However, 26.8% expressed dissatisfaction, indicating that more needs to be done to support cancer prevention for PWID adequately (Table 1).

The data presented in Table 2 provide valuable insights into the awareness and perceived importance of cancer prevention strategies among families of PWID. Most caregivers (41.5%) reported being partially familiar with cancer prevention strategies such as screening programs, lifestyle changes, healthy eating, physical activity, smoking cessation, and sun protection, while 34.1% stated they were completely familiar. However, 7.3% indicated they were completely unfamiliar with such strategies, pointing to a potential gap in public health education regarding cancer prevention, especially in populations caring for PWID.

When asked about the importance of tailored cancer prevention strategies for PWID, the majority (60.9%) expressed that it was “completely important”, with another 29.2% stating it was “partially important”. This response highlights a strong demand for targeted and accessible cancer prevention programs that cater to the specific needs of individuals with ID. Only a small fraction (4.9%) viewed these strategies as unimportant, indicating broad recognition of the need for inclusivity in public health initiatives (Table 2).

Regarding confidence in supporting family members with ID in adopting cancer prevention measures, nearly half of the caregivers (46.3%) felt very confident in their ability to support, and 39% were predominantly confident. These results are encouraging but suggest improvement in equipping caregivers with the necessary knowledge and skills to support PWID in maintaining healthy lifestyles. Notably, 2.4% reported not being confident, pointing to the need for targeted educational interventions (Table 2).

Finally, the data on the perceived effectiveness of current cancer prevention strategies for PWID reveal that most caregivers (58.5%) believe these strategies to be effective, while 14.6% view them as very effective. However, 12.2% are neutral, and 14.6% view the strategy as either partially ineffective or ineffective, which may indicate that caregivers feel existing programs are not sufficiently adapted to the needs of the ID population (Table 2).

The Chi-Square tests’ results for independence revealed no statistically significant difference in caregivers’ knowledge of cancer prevention strategies across caregiver types (parent, sibling, guardian) (χ² = 4.71, *p* = 0.78). Similarly, caregivers have no statistically significant difference in knowledge of cancer prevention strategies based on their caregiver’s ID level (moderate, mild, severe, or profound) (χ² = 11.41, *p* = 0.49). These results imply that knowledge gaps are fairly distributed across caregiver types and levels of intellectual disability, indicating that interventions aimed at improving knowledge could be applied uniformly rather than focusing on specific subgroups.

Through content analysis of the interviews, five key themes were identified: promoting a healthy lifestyle, access to healthcare services, health literacy, psychosocial factors, and social inclusion.

### 3.1. Promoting a Healthy Lifestyle

Caregivers of PWID generally acknowledge the importance of fostering a healthy lifestyle as essential to their well-being. Personal experiences, cultural influences, and systemic support shape their attitudes towards health-promoting behaviours like physical activity, proper nutrition, and smoking cessation. A study by Bog et al. [26] highlights that parents of children with ID often engage in health-promoting activities directly linked to improved quality of life.

*“Of course, we regularly go for walks. In fact, she only wants to walk, so we spend the whole day outside. That way, she avoids doing something else. Everyone knows us, but I know it is good for her”. (Mother)*

However, despite their efforts, many caregivers struggle with consistency, especially regarding physical activity and diet. The challenges stem from balancing family members with ID’s unique needs with the desire to promote healthy habits. The caregiver expressed concern over the difficulty enforcing restrictions, fearing they might complicate PWID lives.

*“Sometimes I have to hide sweets. It is hard for me, but I know it is for his good”. (Mother)*

*“It is already hard enough for him, let alone denying him these sweets too”. (Mother)*

The caregivers expressed a need for support in several areas, including effective communication about the importance of healthy living, guidance on providing health information, and structured programs to promote physical activity tailored to the PWID abilities. Suggested strategies include tailored activities like workshops on healthy eating, exercise planning, and educational programs on sun protection.

### 3.2. Access to Healthcare Services

In line with research by Mohd et al. [27], the overall perception of healthcare access among parents of PWID was positive, as they were encouraged to bring them for regular medical and dental check-ups. Healthcare professionals generally provided equitable treatment and demonstrated supportive attitudes toward PWID. The participants in our study highlighted key challenges related to waitlists, difficulties navigating fragmented healthcare systems, and issues related to mobility and transportation. Most notably, many parents emphasised the importance of effective collaboration with healthcare professionals. When professionals engaged in participatory practices, parents reported easier access to healthcare, while lack of cooperation often led to fragmented services and increased.

*“We have no problems. Our doctor always receives us nicely, listens, and reminds us of the necessary check-ups. She also schedules it for us, so there is no waiting. I felt like an equal team member… we functioned as one family”. (Mother)*

*“We visit professionals and collect all those results, reports, and findings like documentation, but we never received such support to say ‘this is the person we will always return to’”. (Mother)*

Caregivers proposed several strategies to overcome these barriers, including improved coordination and collaboration within healthcare sectors, the provision of mobile cancer screening services, and transportation assistance. They also recommended training for healthcare professionals to familiarise them with participatory practices, which could lead to more effective collaboration and better service continuity.

### 3.3. Health Literacy

Caregivers of PWID often encounter difficulties in understanding health literacy, especially concerning cancer prevention. Although many of them have a reasonable understanding of key cancer-related concepts, particularly if their family member with ID has been diagnosed with cancer, there remains a pressing need for targeted health literacy support [28]. Caregivers with lower levels of health literacy often struggle to comprehend treatment plans and other complex healthcare information, underscoring the importance of tailored educational resources. Furthermore, cognitive and emotional barriers frequently impede their ability to effectively internalise and utilise healthcare information [29].

All caregivers avoid discussing cancer with family members with ID, citing fears that such conversations might frighten them or believing that PWID would not fully understand the topic.

*“We have never talked about cancer. We avoid talking about it. I have cancer, and when I feel bad, I just tell my son that I have a cold”. (Mother)*

Caregivers often rely on internet sources and social media for information, which may lack the reliability and clarity needed for effective healthcare decision making.

*“I started following a Facebook group, which is truly good”. (Mother)*

*“We visit professionals and gather all information. Sometimes it is hard to understand what they want to say, and they do not have time to explain. Then I go to the Internet”. (Mother)*

To address this gap, caregivers suggested creating easy-to-read, picture-based educational materials and step-by-step guidelines for cancer screenings. Visual aids explaining the importance of regular health screenings and caregiver training sessions were also proposed as useful strategies.

### 3.4. Psychosocial Factors and Social Inclusion

Psychosocial factors and social inclusion are critical components of cancer prevention for PWID. Support from family and caregivers plays a pivotal role in encouraging health-promoting behaviours. Inclusive communities help to reduce the barriers that PWID face in accessing healthcare, facilitating better communication and fostering trust between patients and healthcare providers.

*“We meet twice a week, and it means a lot to us. We talk, share experiences, and are there for each other”. (Mother)*

Social inclusion also helps mitigate the feelings of isolation and stigma often associated with reduced healthcare participation. Support groups, where PWID and their caregivers can share experiences and offer mutual encouragement, were considered highly valuable by participants.

The caregivers proposed strategies to enhance social inclusion, such as forming support groups, developing stress management programs tailored to PWID, and creating opportunities for them to participate in community activities that promote health. Ensuring these activities are welcoming, accommodating, and inclusive of PWID was emphasised. Additionally, caregivers stressed the need to include PWID in decision-making processes regarding their healthcare, which could enhance their engagement in health-promoting activities.

## 4. Discussion

The present study explored the challenges and needs related to cancer prevention among caregivers of PWID, revealing critical gaps in awareness, support, and systemic structures. Results from the quantitative and qualitative phases highlighted several key issues, particularly the lack of tailored cancer prevention materials, caregiver knowledge deficits, and systemic barriers to healthcare access.

One of the most significant findings was that the majority of caregivers (75.6%) lacked information specifically related to cancer prevention for PWID. This indicates a crucial gap in the education and resources provided to caregivers. Although general health promotion materials (brochures, posters) are available in healthcare settings, these resources are not tailored to PWID’s unique cognitive and communication needs. Caregivers are left to seek information independently, often relying on unverified or incomplete sources, which can lead to misinformation and suboptimal care practices. This aligns with research by Scherer et al. [30], which also found a lack of accessible resources for PWID. Importantly, this study is compounded by the fact that it did not assess whether caregivers had general knowledge about cancer prevention, raising the question of whether this knowledge deficit is limited to ID-specific concerns or reflects broader health literacy challenges. The lack of differentiation in assessing cancer prevention knowledge may obscure important nuances influencing targeted interventions.

Our study identified numerous barriers to regular cancer screening for PWID. Logistical challenges, such as transportation issues and difficulties navigating the healthcare system, were frequently cited by caregivers. Additionally, many caregivers (63.4%) reported not regularly organising medical screenings. This is concerning, given early detection’s critical role in improving cancer outcomes. In addition, these findings mirror existing literature that highlights systemic barriers to healthcare for PWID. Many of these barriers are not new; they have been documented in prior research, yet little progress has been made to address them [11]. The abovementioned suggests that healthcare systems are not adequately responding to the well-documented needs of this population. Satisfaction with healthcare professional support was mixed, with 26.8% of caregivers expressing dissatisfaction. This dissatisfaction likely stems from the lack of tailored care and insufficient communication between healthcare professionals and caregivers. Inadequate clinical settings and communication challenges, particularly PWID’s difficulty articulating their needs or understanding medical advice, further exacerbate these barriers. This echoes the study of Ervin et al. [31], who identified similar obstacles to healthcare access for PWID. It is common for PWID not to be equally included in decision-making processes as other patients.

In addition to logistical barriers, caregivers reported significant emotional and psychological burdens associated with their caregiving roles. Many expressed stress related to managing the health behaviours of PWID and concerns about their ability to implement lifestyle changes that promote cancer prevention. A recurring theme in the qualitative data was caregivers’ fear of discussing cancer with the individuals they care for, worried that such conversations could cause unnecessary fear or distress. These emotional burdens can also negatively impact caregivers’ health, emotional resilience, and capacity to manage the complexities of care, findings supported by Molassiotis et al. [32].

Caregivers’ reliance on information obtained online or through social media highlights a critical gap in professional support. Many caregivers felt that there was insufficient time during medical visits to discuss cancer prevention or that healthcare professionals did not adequately understand their concerns. This reflects a communication gap, where healthcare providers may lack the skills or time to engage in meaningful discussions with caregivers about cancer prevention. Some research supports this, showing that nurses often avoid open communication with cancer patients due to discomfort or lack of training [33].

All strategies should be inclusive, accessible, and tailored to PWID and their caregivers’ unique needs. The caregivers’ strong demand for tailored cancer prevention strategies (90.3%) suggests that inclusive public health strategies must prioritise the unique health needs of individuals with ID. Caregivers suggest that barriers can be overcome by promoting participatory practices from healthcare professionals. Participatory practices involve professionals actively, including family members, in acquiring information and enabling informed decision making. This also involves utilising the existing strengths and resources of the family and community and helping them access new resources for additional support [34]. Strengthening communication between healthcare workers and caregivers is essential. Specialised training for healthcare professionals focused on PWID and their families’ unique needs would improve interactions and enhance cancer prevention efforts.

The study’s findings emphasise the need for specialised training for healthcare professionals to improve communication with caregivers of PWID. These programs should focus on medical knowledge and developing communication strategies sensitive to the cognitive and emotional needs of PWID and their caregivers. Adequate health education for caregivers can significantly influence cancer prevention and early detection by facilitating easier access to information and better understanding and communication of health information [35]. The fact that most caregivers (80.5%) acknowledged the need for additional training underscores the importance of developing targeted, accessible educational resources that are easy to understand and tailored to the ID population. Bronfenbrenner’s ecological systems theory helps explain the systemic nature of these challenges. The lack of alignment between healthcare professionals and the caregiving environment represents a failure at the mesosystem level, where healthcare providers and families are not sufficiently coordinated to meet the unique needs of individuals with ID. Our data show no significant difference in knowledge about cancer prevention among caregivers based on their relationship to the individual with ID (parent, sibling, guardian). This suggests that family members play a crucial role in shaping health behaviours but are universally under-supported. This reinforces the need for interventions that address the entire caregiving network, ensuring that all caregivers receive the education and support necessary to facilitate cancer prevention, regardless of their role.

The weak-to-moderate association between the severity of ID and caregiver knowledge further indicates that knowledge deficits persist across all caregiver types and levels of ID severity. As such, interventions must target the entire caregiver population and focus on systemic accommodations to address the logistical and educational challenges caregivers face.

Caregivers suggested several practical solutions, such as mobile screening services and better-coordinated care, which could help mitigate some of the barriers identified in this study. Implementing these solutions and offering caregivers psychosocial support could reduce caregiving’s emotional strain and improve health outcomes for PWID. Support groups, as suggested by caregivers, could also serve as valuable platforms for sharing strategies, promoting resilience, and fostering a sense of community.

To advance cancer prevention strategies for people with intellectual disabilities (PWID), a diverse group of European and international stakeholders, including researchers, clinicians, advocacy groups, caregivers, and service users, has come together as part of the ongoing COST Action 21213, titled “Cancer-Understanding Prevention in Intellectual Disabilities”. This interdisciplinary collaboration aims to build capacity to tackle health inequalities and improve access to cancer screening for PWID [21].

### Limitations of the Study

This study has several limitations that should be acknowledged. The sample size was small, and all participants were drawn from a single region, limiting the generalizability of the findings. The study did not account for sociodemographic factors, educational background, or cultural variations that could influence the results, such as general knowledge about cancer or lifestyle habits. Future research should aim to conduct more comprehensive investigations with larger, more diverse samples to understand better the full scope of challenges PWID caregivers face.

## 5. Conclusions

The quantitative findings revealed that most caregivers of PWID (a) had not received information about cancer prevention for PWID, (b) did not regularly coordinate cancer screenings due to identified barriers such as a lack of accessible information, logistical challenges, and emotional strain, and (c) expressed a need for additional training to support their roles in cancer prevention. In addition, the qualitative findings highlighted that caregivers of PWID (a) were engaged in health-promoting activities, (b) had a positive overall perception of healthcare services, (c) had difficulty with health literacy, and (d) needed psychosocial support for participation in cancer prevention for PWID.

To conclude, the study highlights significant gaps in cancer prevention efforts for PWID, particularly in terms of caregiver education and systemic support. Therefore, addressing these gaps will require targeted interventions, including specialised training for healthcare professionals, developing tailored educational resources, and structural changes to improve healthcare access. Moreover, caregivers’ emotional and psychological burdens must not be overlooked, and psychosocial support should be integral to future public health strategies.

## Figures and Tables

**Table 1:** Experiences caregivers regarding cancer prevention in PWID.

Item	n (%)
Have you received any information about cancer prevention in PWID?	
Yes	10 (24.4)
No	31 (75.6)
Additional training for me as a caregiver would help improve cancer prevention in PWID.	
Strongly disagree	0 (0.0)
Disagree	0 (0.0)
Neutral	8 (19.5)
Agree	20 (48.8)
Strongly agree	13 (31.7)
Do you plan and coordinate PWID participation in organised cancer screening programs (e.g., mammography, colonoscopy, gynaecological examination)?	
Yes	15 (36.6)
No	26 (63.4)
How often do you encounter difficulties when PWID performs regular screening (mammography, colonoscopy, gynaecological examination)?	
Always	5 (12.2)
Often	11 (26.8)
Rarely	5 (12.2)
Sometimes	10 (24.4)
Never	10 (24.4)
How satisfied are you with the support received from healthcare professionals regarding cancer prevention in PWID?	
Completely satisfied	4 (9.8)
Satisfied	9 (22.0)
Neutral	17 (41.5)
Dissatisfied	11 (26.8)
Completely dissatisfied	0 (0.0)

**Table 2:** Awareness of cancer prevention strategies in PWID.

Item	n (%)
How familiar are you with cancer prevention strategies (screening programs, lifestyle changes: healthy eating, physical activity, smoking cessation, sun protection)?	
Completely familiar	14 (34.1)
Partially familiar	17 (41.5)
Neutral	4 (9.8)
Partially unfamiliar	3 (7.3)
Completely unfamiliar	3 (7.3)
How important would it be to you if cancer prevention strategies were adapted to PWID?	
Completely important	25 (60.9)
Partially important	12 (29.3)
Neutral	0 (0.0)
Partially unimportant	2 (4.9)
Completely unimportant	2 (4.9)
How confident are you in supporting a PWID in implementing healthy lifestyle strategies (strategies) to prevent cancer (screening programs, lifestyle changes: healthy eating, physical activity, smoking cessation, sun protection)?	
Very confident	19 (46.3)
Predominantly confident	16 (39.0)
Fairly confident	1 (2.4)
Only slightly confident	4 (9.8)
Not at all confident	1 (2.4)
To what extent do you believe the strategies you apply to prevent cancer are effective for PWID?	
Very effective	6 (14.6)
Effective	24 (58.5)
Neutral	5 (12.2)
Partially ineffective	5 (12.2)
Ineffective	1 (2.4)

## Data Availability

The data presented in this study are available from the corresponding authors upon reasonable request.

## References

[1] World Health Organization (2024). Global Cancer Burden Growing, Amidst Mounting Need for Services. https://www.who.int/news/item/01-02-2024-global-cancer-burden-growing--amidst-mounting-need-for-services.

[2] American Cancer Society (2024). Cancer Facts and Figures 2024. https://www.cancer.org/content/dam/cancer-org/research/cancer-facts-and-statistics/annual-cancer-facts-and-figures/2024/2024-cancer-facts-and-figures-acs.pdf.

[3] Siegel R.L., Giaquinto A.N., Jemal A. (2024). Cancer statistics, 2024. CA Cancer J. Clin..

[4] Schalock R.L., Luckasson R., Tassé M.J. (2021). Intellectual Disability: Definition, Diagnosis, Classification, and Systems of Supports.

[5] Breau G., Thorne S., Baumbusch J., Hislop T.G., Kazanjian A. (2023). Family physicians’ and trainees’ experiences regarding cancer screening with patients with intellectual disability: An interpretive description study. J. Intellect. Disabil..

[6] Chan D.N.S., Law B.M.H., Au D.W.H., So W.K.W., Fan N. (2022). A systematic review of the barriers and facilitators influencing the cancer screening behaviour among people with intellectual disabilities. Cancer Epidemiol..

[7] Power R., David M., Strnadová I., Touyz L., Basckin C., Loblinzk J., Jolly H., Kennedy E., Ussher J., Sweeney S. (2024). Cervical screening participation and access facilitators and barriers for people with intellectual disability: A systematic review and meta-analysis. Front. Psychiatry.

[8] Cuypers M., Schalk B.W.M., Boonman A.J.N., Naaldenberg J., Leusink G.L. (2022). Cancer-related mortality among people with intellectual disabilities: A nationwide population-based cohort study. Cancer.

[9] Cuypers M., Cairns D., Robb K.A. (2024). Identifying the deficits in cancer care for people with intellectual disabilities. BMJ Oncol..

[10] Sonderlund A.L., Baygi F., Sondergaard J., Thilsing T. (2024). Advancing health equity for populations with intellectual disabilities: A systematic review of facilitators and barriers to implementation of health checks and screening. SSM-Health Syst..

[11] Shady K., Phillips S., Newman S. (2022). Barriers and Facilitators to Healthcare Access in Adults with Intellectual and Developmental Disorders and Communication Difficulties: An Integrative Review. Rev. J. Autism Dev. Disord..

[12] Krahn G., Fox M.H., Campbell V.A., Ramon I., Jesien G. (2010). Developing a health surveillance system for people with intellectual disabilities in the United States. J. Policy Pract. Intellect..

[13] Vaitsiakhovich N., Landes S.D. (2023). The association between the patient protection and Affordable Care Act and healthcare affordability among US adults with intellectual disability. J. Intellect. Disabil. Res..

[14] Sanderson K.A., Bumble J.L., Kuntz E.M. (2020). Meeting the daily needs of adults with IDD: The importance of informal supports. Int. Rev. Res. Dev. Disabil..

[15] Factor R.S., Estabillo J.A., Bennett G., Goodall E. (2024). Parent Support for Mental Healthcare of Individuals with Intellectual Disabilities. The Palgrave Encyclopedia of Disability.

[16] Crawford M. (2020). Ecological Systems theory: Exploring the development of the theoretical framework as conceived by Bronfenbrenner. J. Public Health Issues Pract..

[17] Raymond M., Simonetta M.A. (2023). The cancer caregiver lived experience: Supporting and advocating for patients with cancer throughout the care continuum. JCO Oncol. Pract..

[18] Moreland S. (2023). Bridging the Gap in Healthcare Information Access For Caregivers. J. Ahima.

[19] Riches V.C., O’Brien P., Manokara V., Mueller A. (2023). A study of caregiver support services: Perspectives of family caregivers of persons with intellectual disabilities in Singapore. J. Policy Pract. Intellect..

[20] Burke M.M., Lulinski A., Jones J., Gallus K. (2018). A review of supports and services for adults with intellectual and developmental disabilities (IDD) and their families in the United States: Past and present contexts impacting future research, practice and policy. Int. Rev. Res. Dev. Disabil..

[21] Vuković V., Banda A., Carneiro L., Dogan S., Knapp P., McMahon M., Milutinovic D., Soylar P., Sykes K., Tosun B. (2023). The importance of cancer prevention policies to inform and guide preventative and screening measures for people with intellectual disabilities: The COST project “Cancer-Understanding Prevention in Intellectual Disabilities”. J. Intellect. Disabil..

[22] Creswell J.W., Creswell J.D. (2014). Research Design: Qualitative, Quantitative, and Mixed Methods Approaches.

[23] Tong A., Sainsbury P., Craig J. (2007). Consolidated criteria for reporting qualitative research (COREQ): A 32-item checklist for interviews and focus groups. Int. J. Qual. Health Care.

[24] Hennink M., Kaiser B.N. (2022). Sample sizes for saturation in qualitative research: A systematic review of empirical tests. Soc. Sci. Med..

[25] Braun V., Clarke V. (2019). Reflecting on reflexive thematic analysis. Qual. Res. Sport Exerc. Health.

[26] Jeoung B. (2022). Quality of life and health-promoting lifestyles for parents of children with intellectual and developmental disabilities. J. Exerc. Rehabil..

[27] Mohd F.N., Said A.H., Mat Naji A.S. (2023). Perceptions Toward Healthcare and Dental Care Services among Parents and Caretakers of People with Intellectual Disability (PWID)-A Questionnaire Study. Int. Soc. Prev. Community Dent..

[28] Keith K.E., Wadhwa A., York J., Fazeli P.L., Bhatia S., Landier W. (2023). Health Literacy in Parents of Children Newly Diagnosed With Cancer and Comprehension of Key Concepts Related to Their Child’s Care. J. Pediatr. Hematol. Oncol. Nurs..

[29] Tan C.E., Lau S.C.D., Abdul Latiff Z., Lee C.C., The K.H., Mohd Sidik S. (2024). Parents of children with cancer require health literacy support to meet their information needs. Health Inf. Libr. J..

[30] Scherer N., Wiseman P., Watson N., Brunner R., Cullingworth J., Hameed S., Pearson C., Shakespeare T. (2023). ‘Do they ever think about people like us?’: The experiences of people with learning disabilities in England and Scotland during the COVID-19 pandemic. Crit. Soc. Policy.

[31] Ervin D.A., Hennen B., Merrick J., Morad M. (2014). Healthcare for persons with intellectual and developmental disability in the community. Front. Public Health.

[32] Molassiotis A., Wang M. (2022). Understanding and Supporting Informal Cancer Caregivers. Curr. Treat. Options Oncol..

[33] Kelsey S. (2005). Improving nurse communication skills with the cancer patient. Cancer Nurs. Pract..

[34] Dunst C.J., Trivette C.M., Hamby D.W. (2007). Meta-analysis of family-centered helpgiving practices research. Ment. Retard. Dev. Disabil. Res. Rev..

[35] Moore C., Hassett D., Dunne S. (2021). Health literacy in cancer caregivers: A systematic review. J. Cancer Surviv..

